# Lead and Retinal Function: Low Prenatal Exposure Suggests Future Abnormality

**Published:** 2008-05

**Authors:** Valerie J. Brown

The harmful effects of low-level lead exposure on the development of cognitive, auditory, and visual-motor functions in children are well documented, but few studies have focused on the effects of low-level lead exposure specifically on retinal and visual functions. A rodent study reported this month provides new evidence that low levels of gestational lead exposure (GLE) can cause permanent retinal abnormalities in adult offspring and supports existing evidence that prenatal lead exposure can affect retinal development and function in humans even at blood lead levels below 10 μg/dL, the level of concern identified by the Centers for Disease Control and Prevention **[*EHP* 116:618–625; Fox et al.]**.

Researchers exposed two groups of female rats to lead in drinking water. A GLE group was exposed from 2 weeks before breeding through post-natal day 10 and a postnatal lead exposure (PLE) group from delivery through pup weaning. Each group was divided into four subgroups that were exposed to varying doses of lead (0 ppm [control], 27 ppm, 55 ppm, or 109 ppm). Maternal blood lead levels in the GLE rats were similar to those observed in human mothers whose children had experienced prenatal lead exposure.

The researchers included the PLE group for comparison because in previous studies PLE caused rod-selective cell death (apoptosis) and decreased retinal electrical activity as measured by an electroretinogram (ERG). Previous studies have demonstrated that postnatal blood lead levels greater than 20 μg/dL in lead-exposed humans and animals cause decreased ERG amplitudes (“subnormality”), whereas children exposed gestationally or postnatally who had blood lead levels of 6–16 μg/dL exhibited increased ERG amplitudes (“supernormality”).

In the current study, when offspring in the GLE group reached adulthood, the researchers tested the animals’ rod retinal function using an ERG, then counted the number of rods and cones in the rats’ eyes. (Rods are photoreceptors active in low light; cones provide color vision.) Last, the researchers measured the synthesis of retinal dopamine, a neurotransmitter that regulates several retinal processes, including cell survival and eye growth.

Results from the GLE group showed an inverted dose–response curve typical of lead neurotoxicity. In adult offspring, low and moderate levels of GLE produced supernormal scoptic ERGs, increased numbers of rod cells and rod bipolar cells, and decreased dopamine synthesis and release in the absence of retinal injury, potentially heightening the likelihood of late-onset retinal degeneration. High levels of GLE produced opposite results.

The authors note that the scotopic supernormal ERG can be a noninvasive biomarker of GLE. They also interpret the inverted dose–response curve as a sign of the retina’s long-term vulnerability to low-level lead exposure during gestation. They acknowledge their data may raise complex issues for risk assessment and indicate that dose-and state-dependent effects are important in neurotoxicity risk assessment.

## Figures and Tables

**Figure:**
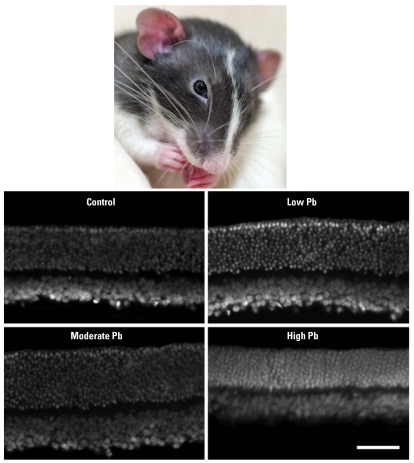
Thickening of the adult rat retina following low or moderate exposure to lead reflects changes in cell numbers.

